# 

**Published:** 1861-12

**Authors:** 


New Series, Vol. 4, No. 12. Whole Series, Vol. 18, No. 12.

### THE CHICAGO MEDICAL JOURNAL.

DANIEL BRAINARD, M. D.,

J. ADAMS ALLEN, M. D.,

Editors and Proprietors.

DECEMBER, 1861.

CHICAGO:
WM. PIGOTT, BOOK AND JOB PRINTER, 130 CLARK STREET.
1861.

